# Crystal structure and Hirshfeld surface analysis of 2,4-di­amino-6-methyl-1,3,5-triazin-1-ium tri­chloro­acetate monohydrate

**DOI:** 10.1107/S2056989018008307

**Published:** 2018-06-12

**Authors:** Ramalingam Sangeetha, Kasthuri Balasubramani, Kaliyaperumal Thanigaimani, Savaridasson Jose Kavitha

**Affiliations:** a Department of Chemistry, Governemnt Arts College (Autonomous), Karur 639 005, Tamil Nadu, India; b Department of Chemistry, Government Arts College, Thiruchirappalli 620 022, Tamil Nadu, India; c Department of Chemistry, Mother Teresa Womens University, Kodaikanal 624 102, Tamil Nadu, India

**Keywords:** crystal structure, triazinium cation, tri­chloro­acetate anion, three-dimensional hydrogen-bonded supra­molecular framework, Hirshfeld surface analysis

## Abstract

The asymmetric unit of the title mol­ecular salt, C_4_H_8_N_5_^+^·C_2_Cl_3_O_2_^−^·H_2_O, coomprises a 2,4-di­amino-6-methyl-1,3,5-triazin-1-ium cation, a tri­chloro­acetate anion and a water mol­ecule of solvation. The protonated N atom of the cation forms a hydrogen bond with a carboxyl O atom of the anion, which also acts as a hydrogen-atom acceptor with the water mol­ecule. The cations form centrosymmetric dimeric units through *R*_2_^2^(8) N—H⋯N bond pairs and are extended into zigzag chains along the *c*-axis direction, also through similar cyclic *R*_2_^2^(8) dual N—H⋯N hydrogen-bonding inter­actions. The water mol­ecule acts as a dual acceptor forming N—H⋯O hydrogen bonds between the amine groups of the cations, forming cyclic *R*_2_^3^(8) motifs. The second H atom of the water mol­ecule also acts as a donor in an O—H⋯O hydrogen bond with the second carboxyl O atom, linking the chains along the *b*-axis direction. These interactions give rise to an overall three-dimensional supra­molecular structure. A Hirshfeld surface analysis was employed in order to study the inter­molecular inter­actions.

## Abstract 2

In the crystal structure, the cations form hydrogen-bonded zigzag chains through centrosymmetric cyclic 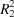
(8) N—H⋯N associations while the water mol­ecule acts as a double acceptor, linking the cations of the chain peripherally through amine N—H⋯O hydrogen bonds, closing cyclic 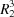
(8) motifs, and as a double O—H⋯O donor, linking the anions, giving an overall three-dimensional structure.

## Chemical context   

Triazine heterocyclic π-conjugated structures are attractive owing to the chemical flexiblity of their systems and have many applications in medicinal chemistry, materials science and organic synthesis (Boesveld & Lappert, 1997 ▸; Boesveld *et al.*, 1999 ▸; Reid *et al.*, 2011 ▸). 1,3,5-Triazine derivatives represent an important class of compounds because of their potential to be biologically active. They are known to be anti-protozoal agents (Baliani *et al.*, 2005 ▸), anti­cancer agents (Menicagli *et al.*, 2004 ▸), estrogen receptor modulators (Henke *et al.*, 2002 ▸), anti-malarials (Agarwal *et al.*, 2005 ▸), cyclin-dependent kinase modulators (Kuo *et al.*, 2005 ▸) and anti-microbial agents (Koc *et al.*, 2010 ▸). These compounds still continue to be the object of considerable inter­est mainly because of their applications in various fields, including the production of herbicides and polymer photostabilizers. Triazine derivatives have been used as building blocks for subtle chemical architectures comprising organic–inorganic hybrid frameworks (Ma­thias *et al.*, 1994 ▸; Zerkowski & Whitesides, 1994 ▸; MacDonald & Whitesides, 1994 ▸; Guru Row, 1999 ▸; Krische & Lehn, 2000 ▸; Sherrington & Taskinen, 2001 ▸). In these approaches, interplay between mol­ecules is achieved by using diverse styles of non-covalent inter­actions, which include hydrogen bonds or ionic, hydro­phobic, van der Waals or dispersive forces. Herein, the crystal structure of the title compound salt, 2,4-di­amino-6-methyl-1,3,5-triazine-5-ium tri­chloro­acetate monohydrate is reported. Hirshfeld surface analysis and 2D fingerprint plots were employed in order to qu­antify the contributions of the various inter­molecular inter­actions present in the structure.
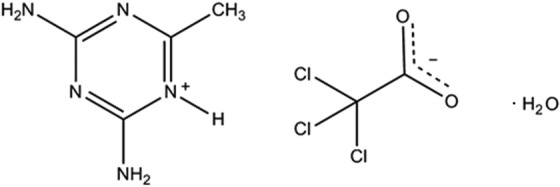


## Structural commentary   

**Figure 1:**
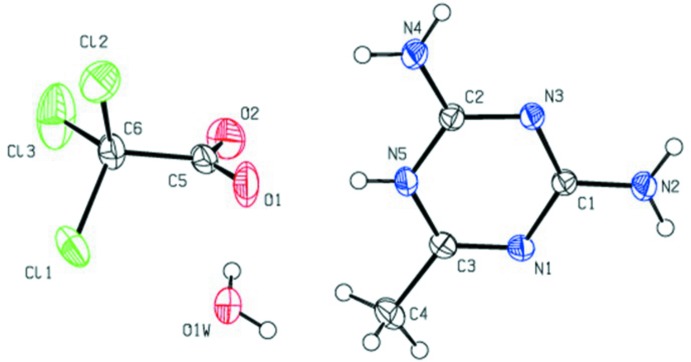
The mol­ecular structure and atom-numbering scheme for the title salt, with displacement ellipsoids drawn at the 40% probability level.

The mol­ecular structure with atomic numbering scheme is shown in Fig. 1 ▸. The asymmetric unit comprises a 2,4-di­amino-6-methyl-1,3,5-triazine-5-ium cation, a tri­chloro­acetate anion and a water mol­ecule of solvation (O1*W*). Proton transfer occurs from one of the carb­oxy­lic acid oxygen atoms (O1) to atom N5 of the cation, with a resulting N5—H1*N*5⋯O1 hydrogen bond [2.652 (3) Å, Table 1 ▸]. The water mol­ecule is also hydrogen bonded to atom O1 [2.835 (3) Å]. The proton transfer to the cation results in a widening of the C3—N5—C2 bond angle of the triazinium ring to 119.06 (19)°, compared to the comparative angle found in neutral 2,4-di­amino-6-methyl-1,3,5-triazine [114.4 (7)°; Aoki *et al.*, 1994 ▸]. The C—O bond distances within the carboxyl group of the tri­chloro­acetate anion are 1.212 (3) and 1.251 (3) Å.

## Supra­molecular features   

**Figure 2:**
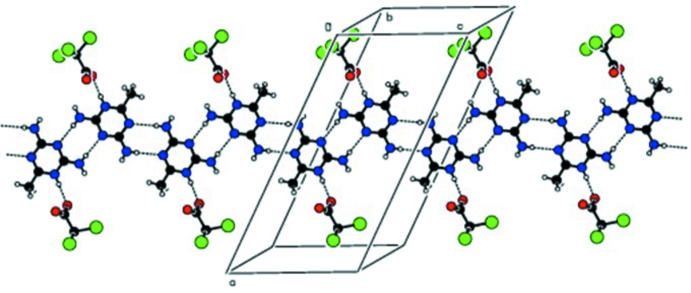
A packing view showing the centrosymmetric N—H⋯N hydrogen-bonded cation pairs with TCA anions, extending into chains along the *c*-axis direction. Water mol­ecules are omitted.

**Figure 3:**
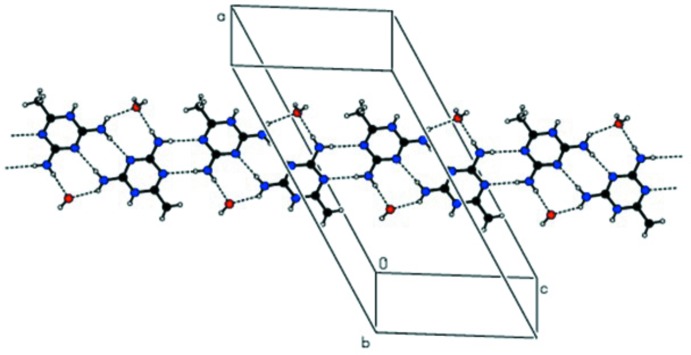
Another view of the extended chains with the TCA anions omitted, showing the *DADA* array and the participation of the water mol­ecules in hydrogen bonding.

**Figure 4:**
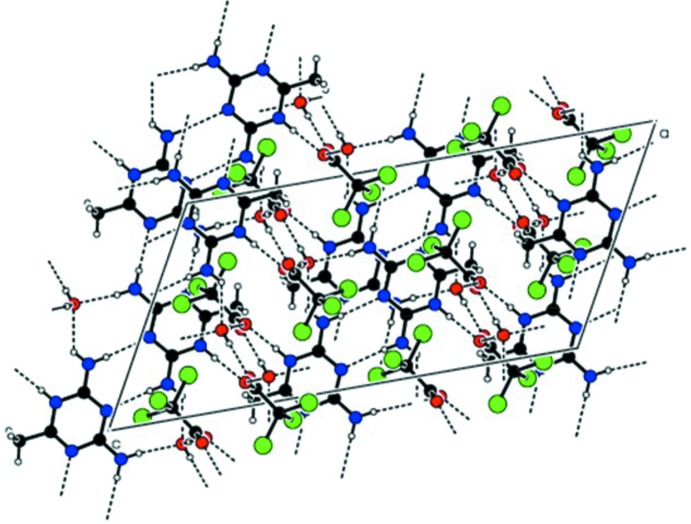
An overall view of the three-dimensional hydrogen-bonded supra­molecular structure.

In the crystal, pairs of 2,4-di­amino-6-methyl-1,3,5-triazine-5-ium cations associate through lateral centrosymmetric inter­actions *via* N2—H2*N*2⋯N1^iii^ and N4—H2*N*4⋯N3^iv^ hydrogen bonds (Table 1 ▸) with cyclic 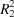
(8) graph-set motifs. These inter­actions result in the formation of zigzag chains extending along the *c*-axis direction (Fig. 2 ▸). The cations in the chains are further linked through amine N2—H1*N*2⋯O1*W* ^ii^ and N4—H1*N*4⋯O1*W* ^v^ hydrogen bonds in 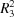
(8) motifs (Fig. 3 ▸), producing a complementary *DADA* (*D* = donor and *A* = acceptor) hydrogen-bonded array with an 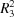
(8), 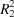
(8), 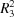
(8) graph-set motif sequence (Fig. 3 ▸). The water mol­ecule acts as a donor to form a second O1*W*—H2*O*2⋯O2^vi^ hydrogen bond, which together with the O1*W*—H1*O*1⋯O1 hydrogen-bond sequence links the tri­chloro­acetate anions into chains along the *b*-axis direction. Overall, a three-dimensional supra­molecular structure is generated (Fig. 4 ▸).

## Hirshfeld surface analysis   

**Figure 5:**
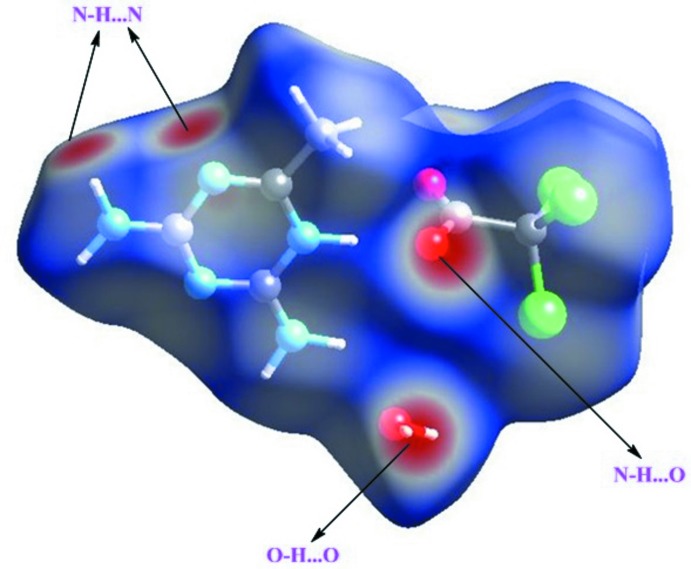
The three-dimensional Hirshfeld surface of the title compound

**Figure 6:**
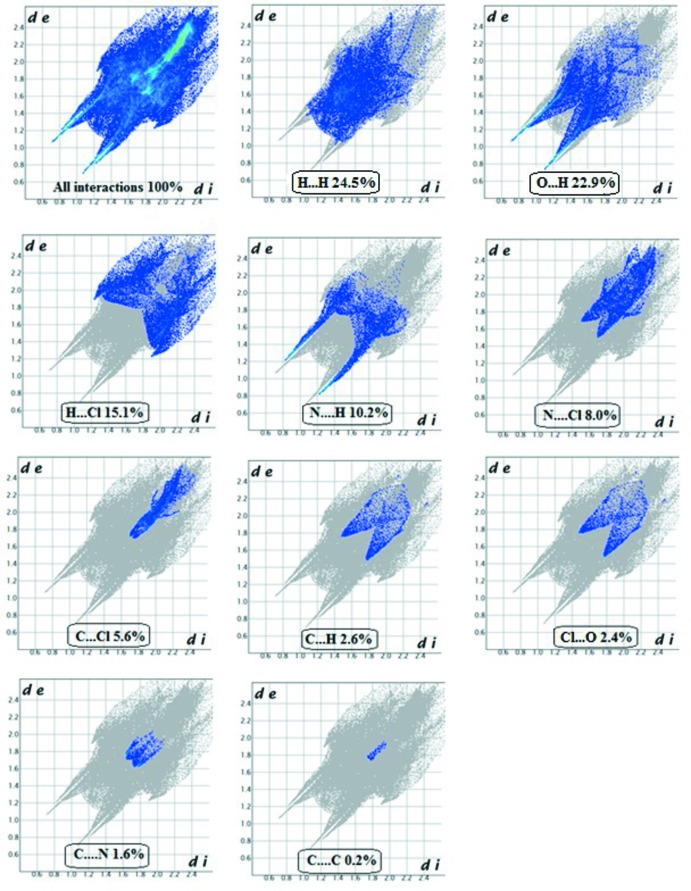
Two-dimensional fingerprint plots for the title compound

Hirshfeld surface analysis (Spackman & Jayatilaka, 2009 ▸) and 2D fingerprint plots are useful tools for describing the surface characteristics of the crystal structure and were generated using CrystalExplorer 3.0 (Wolff *et al.*, 2012 ▸). The normalized contact distance (*d*_norm_) is based on the distances from the nearest atom inside (*d*_i_) and outside (*d*_e_) the surface. The 3D *d*_norm_ surface of the title compound is shown in Fig. 5 ▸. The red points represent closer contacts and negative *d*_norm_ values on the surface corresponding to the N—H⋯O, N—H⋯N and O—H⋯O inter­actions. Two-dimensional fingerprint plots are shown in Fig. 6 ▸. H⋯H inter­actions (24.5%) are present as a major contributor while H⋯O/O⋯H (22.9%), N⋯H/H⋯N (10.2%), H⋯Cl (15.1%) N⋯H (10.2%), N⋯Cl (8.0%), C⋯Cl (5.6%), C⋯H (2.6%), Cl⋯O (2.4%), C⋯N (1.6%) and C⋯C (0.2%) contacts also make significant contributions to the Hirshfeld surface.

## Database survey   

A search of the Cambridge Structural Database (Version 5.37, update February 2016; Groom *et al.*, 2016 ▸) for 2,4-di­amino-6-methyl-1,3,5-triazine yielded 22 structures of proton-transfer salts with carb­oxy­lic acids: AZUYUQ (with tetra­fluoro­boric acid; Gomathi & Mu­thiah, 2011 ▸); CICZUK (with tri­fluoro­acetic acid; Perpétuo & Janczak, 2007 ▸); GIMRIE (with hydrogen chloride; Portalone & Colapietro, 2007 ▸); KUSQEV (with hydrogen chloride; Qian & Huang, 2010 ▸); LUGGEB (with 3,5-di­hydroxy­benzoic acid; Xiao *et al.*, 2014 ▸); NAGLIR (with dimesyl­amide; Wijaya *et al.*, 2004 ▸); QUWXAI (with 2-carb­oxy­benzoic acid), QUWXEM [with (*Z*)-2-carb­oxy­ethene-1-carb­oxy­lic acid] and QUWXIQ (with 3-hy­droxy­pyridine-2-carb­oxy­lic acid) (Thanigaimani *et al.*, 2010 ▸); ROGPIN [with oxalic acid (methanol clathrate)], ROGPOT [with malonic acid (tetra­hydrate clathrate)], ROGPUZ [with succinic acid (clathrate)], ROGQAG [with acetyl­enedi­carb­oxy­lic acid (monohydrate clathrate)], ROGQEK [glutaric acid (clathrate)], ROGQIO [thio­diglycolic acid(clathrate)], ROGQOU [diglycolic acid (monohydrate clathrate)], ROMZOJ [fumaric acid (clathrate)] (Delori *et al.*, 2008 ▸); SOLTIX (with nitric acid; Fan *et al.*, 2009 ▸); YODCAX (with 2,3,5,6-tetra­fluoro­terephthalic acid; Wang *et al.*, 2014 ▸); ZAQJEI (with oxalic acid; Narimani & Yamin, 2012 ▸); ZUDSOI [with 6-chloro­uracil-1-ide (*N*,*N*-di­methyl­acetamide solvate)], ZUDSUO [with 6-chloro­uracil-1-ide (*N*,*N*-di­methyl­formamide solvate monohydrate)] (Gerhardt & Egert, 2015 ▸).

## Synthesis and crystallization   

The title compound was prepared by mixing a hot methano­lic solution (20 ml) of 2,4-di­amino-6-methyl-1,3,5-triazine (1.25 mg) and an aqueous solution (10 ml) of tri­chloro­acetic acid (1.63 mg) in a 1:1 molar ratio. The reaction mixture was warmed over a water bath for a few minutes. The resultant solution was then allowed to cool slowly at room temperature. After a few days, colourless block-shaped crystals of the title compound were separated out.

## Refinement   

Crystal data, data collection and structure refinement details are summarized in Table 2 ▸. The C- and N-bound H atoms were placed in calculated positions and were included in the refinement in the riding-model approximation with C—H = 0.96 Å and N—H = 0.86 Å (NH, NH_2_), with *U*_iso_(H) set to 1.2*U*_eq_(C,N). The water-bound H atoms were located in a difference-Fourier map and were freely refined [O—H = 0.78 (4) and 0.86 (4) Å].

## Supplementary Material

Crystal structure: contains datablock(s) global, I. DOI: 10.1107/S2056989018008307/zs2400sup1.cif

Structure factors: contains datablock(s) I. DOI: 10.1107/S2056989018008307/zs2400Isup2.hkl

Supporting information file. DOI: 10.1107/S2056989018008307/zs2400Isup3.cml

CCDC reference: 1587723

Additional supporting information:  crystallographic information; 3D view; checkCIF report

## Figures and Tables

**Table 1:** Hydrogen-bond geometry (Å, °)

*D*—H⋯*A*	*D*—H	H⋯*A*	*D*⋯*A*	*D*—H⋯*A*
N5—H1*N*5⋯O1^i^	0.86	1.79	2.652 (3)	178
N2—H1*N*2⋯O1*W* ^ii^	0.86	2.03	2.886 (3)	174
N2—H2*N*2⋯N1^iii^	0.86	2.21	3.071 (3)	174
N4—H2*N*4⋯N3^iv^	0.86	2.18	3.034 (3)	173
N4—H1*N*4⋯O1*W* ^v^	0.86	2.22	2.834 (3)	128
O1*W*—H1*O*1⋯O1	0.86 (4)	1.97 (4)	2.835 (3)	176 (3)
O1*W*—H2*O*2⋯O2^vi^	0.78 (4)	1.97 (4)	2.741 (3)	173 (3)

Symmetry codes: (i) 
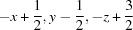
; (ii) 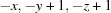; (iii) 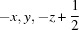; (iv) 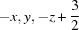; (v) 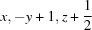; (vi) 
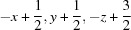
.

**Table 2:** Experimental details

Crystal data	
Chemical formula	C_4_H_8_N_5_^+^·C_2_Cl_3_O_2_^−^·H_2_O
*M*_r_	306.54
Crystal system, space group	Monoclinic, *C*2/*c*
Temperature (K)	293
*a*, *b*, *c* (Å)	21.7056 (18), 11.9074 (9), 10.9562 (6)
β (°)	119.084 (5)
*V* (Å^3^)	2474.7 (3)
*Z*	8
Radiation type	Mo *K*α
μ (mm^−1^)	0.75
Crystal size (mm)	0.35 × 0.30 × 0.30

Data collection	
Diffractometer	Bruker Kappa APEXII CCD
Absorption correction	Multi-scan (*SADABS*; Bruker, 2004 ▸)
*T*_min_, *T*_max_	0.781, 0.807
No. of measured, independent and observed [*I* > 2σ(*I*)] reflections	9801, 3027, 2280
*R*_int_	0.027
(sin θ/λ)_max_ (Å^−1^)	0.667

Refinement	
*R*[*F* ^2^ > 2σ(*F* ^2^)], *wR*(*F* ^2^), *S*	0.046, 0.159, 1.01
No. of reflections	3027
No. of parameters	163
H-atom treatment	H atoms treated by a mixture of independent and constrained refinement
Δρ_max_, Δρ_min_ (e Å^−3^)	0.68, −0.59

Computer programs: *APEX2*, *SAINT* and *XPREP* (Bruker, 2004 ▸), *SHELXS97* and *SHELXL97* (Sheldrick, 2008 ▸) and *PLATON* (Spek, 2009 ▸).

## supplementary crystallographic information

### Crystal data

**Table:**

C_4_H_8_N_5_^+^·C_2_Cl_3_O_2_^−^·H_2_O	*F*(000) = 1248
*M**_r_* = 306.54	*D*_x_ = 1.645 Mg m^−^^3^*D*_m_ = 1.646 Mg m^−^^3^*D*_m_ measured by Not Measured
Monoclinic, *C*2/*c*	Mo *K*α radiation, λ = 0.71073 Å
Hall symbol: -C 2yc	Cell parameters from 3519 reflections
*a* = 21.7056 (18) Å	θ = 6.6–56.0°
*b* = 11.9074 (9) Å	µ = 0.75 mm^−^^1^
*c* = 10.9562 (6) Å	*T* = 293 K
β = 119.084 (5)°	Block, colorless
*V* = 2474.7 (3) Å^3^	0.35 × 0.30 × 0.30 mm
*Z* = 8	

### Data collection

**Table:**

Bruker Kappa APEXII CCD diffractometer	3027 independent reflections
Radiation source: fine-focus sealed tube	2280 reflections with *I* > 2σ(*I*)
Graphite monochromator	*R*_int_ = 0.027
Detector resolution: 18.4 pixels mm^-1^	θ_max_ = 28.3°, θ_min_ = 3.3°
ω and φ scan	*h* = −27→28
Absorption correction: multi-scan (SADABS; Bruker, 2004)	*k* = −15→14
*T*_min_ = 0.781, *T*_max_ = 0.807	*l* = −14→8
9801 measured reflections	

### Refinement

**Table:**

Refinement on *F*^2^	Primary atom site location: structure-invariant direct methods
Least-squares matrix: full	Secondary atom site location: difference Fourier map
*R*[*F*^2^ > 2σ(*F*^2^)] = 0.046	Hydrogen site location: inferred from neighbouring sites
*wR*(*F*^2^) = 0.159	H atoms treated by a mixture of independent and constrained refinement
*S* = 1.01	*w* = 1/[σ^2^(*F*_o_^2^) + (0.0984*P*)^2^ + 1.9287*P*] where *P* = (*F*_o_^2^ + 2*F*_c_^2^)/3
3027 reflections	(Δ/σ)_max_ = 0.001
163 parameters	Δρ_max_ = 0.68 e Å^−^^3^
0 restraints	Δρ_min_ = −0.59 e Å^−^^3^

### Special details

**Table:**

Geometry. Bond distances, angles etc. have been calculated using the rounded fractional coordinates. All su's are estimated from the variances of the (full) variance-covariance matrix. The cell esds are taken into account in the estimation of distances, angles and torsion angles
Refinement. Refinement on F^2^ for ALL reflections except those flagged by the user for potential systematic errors. Weighted R-factors wR and all goodnesses of fit S are based on F^2^, conventional R-factors R are based on F, with F set to zero for negative F^2^. The observed criterion of F^2^ > 2sigma(F^2^) is used only for calculating -R-factor-obs etc. and is not relevant to the choice of reflections for refinement. R-factors based on F^2^ are statistically about twice as large as those based on F, and R-factors based on ALL data will be even larger.

### Fractional atomic coordinates and isotropic or equivalent isotropic displacement parameters (Å^2^)

**Table:**

	*x*	*y*	*z*	*U*_iso_*/*U*_eq_	
Cl1	0.06709 (3)	0.61615 (7)	0.46638 (9)	0.0614 (3)	
Cl2	0.13757 (4)	0.58198 (9)	0.30457 (7)	0.0653 (3)	
Cl3	0.08995 (5)	0.39459 (7)	0.40021 (13)	0.0922 (4)	
N1	0.06762 (9)	0.13611 (16)	0.43697 (17)	0.0272 (5)	
N2	−0.05081 (9)	0.14541 (17)	0.35783 (18)	0.0318 (5)	
N3	0.01987 (9)	0.13451 (15)	0.59533 (17)	0.0260 (5)	
N4	0.09746 (10)	0.12278 (18)	0.82990 (18)	0.0361 (6)	
N5	0.14168 (9)	0.13060 (16)	0.67706 (18)	0.0293 (5)	
O1	0.22699 (10)	0.62450 (18)	0.6137 (2)	0.0559 (7)	
C1	0.01284 (10)	0.13863 (17)	0.46625 (19)	0.0242 (5)	
O2	0.22226 (11)	0.4396 (2)	0.6332 (2)	0.0575 (7)	
C2	0.08544 (10)	0.13021 (17)	0.7000 (2)	0.0251 (5)	
C3	0.13012 (11)	0.13324 (18)	0.5439 (2)	0.0274 (6)	
C4	0.19386 (13)	0.1349 (3)	0.5269 (3)	0.0442 (8)	
O1W	0.17844 (9)	0.8439 (2)	0.6222 (2)	0.0433 (6)	
C5	0.19921 (11)	0.5296 (2)	0.5782 (2)	0.0362 (7)	
C6	0.12614 (11)	0.5291 (2)	0.4432 (2)	0.0374 (6)	
H1N5	0.18400	0.12920	0.74610	0.0350*	
H1N2	−0.08710	0.14710	0.37000	0.0380*	
H2N2	−0.05620	0.14810	0.27480	0.0380*	
H2N4	0.06270	0.12070	0.84680	0.0430*	
H4A	0.18040	0.12240	0.43060	0.0660*	
H4B	0.22570	0.07680	0.58310	0.0660*	
H4C	0.21670	0.20650	0.55590	0.0660*	
H1N4	0.14000	0.12000	0.89770	0.0430*	
H1O1	0.1914 (16)	0.776 (3)	0.619 (3)	0.045 (8)*	
H2O2	0.205 (2)	0.868 (3)	0.695 (4)	0.068 (12)*	

### Atomic displacement parameters (Å^2^)

**Table:**

	*U*^11^	*U*^22^	*U*^33^	*U*^12^	*U*^13^	*U*^23^
Cl1	0.0282 (3)	0.0761 (6)	0.0743 (5)	0.0099 (3)	0.0205 (3)	−0.0050 (4)
Cl2	0.0627 (5)	0.0932 (7)	0.0349 (4)	0.0076 (4)	0.0198 (3)	0.0068 (3)
Cl3	0.0671 (6)	0.0422 (5)	0.1127 (8)	−0.0123 (4)	0.0008 (5)	−0.0058 (5)
N1	0.0247 (8)	0.0366 (10)	0.0229 (8)	0.0001 (7)	0.0136 (7)	−0.0006 (7)
N2	0.0231 (8)	0.0491 (11)	0.0224 (8)	0.0025 (8)	0.0104 (7)	0.0010 (8)
N3	0.0217 (8)	0.0349 (10)	0.0214 (8)	0.0014 (6)	0.0105 (7)	0.0000 (6)
N4	0.0270 (9)	0.0596 (13)	0.0197 (8)	0.0012 (8)	0.0097 (7)	0.0017 (8)
N5	0.0192 (8)	0.0425 (11)	0.0236 (8)	0.0013 (7)	0.0084 (7)	0.0013 (7)
O1	0.0277 (9)	0.0623 (14)	0.0515 (11)	−0.0021 (8)	−0.0014 (8)	−0.0080 (9)
C1	0.0228 (9)	0.0270 (10)	0.0224 (9)	0.0003 (7)	0.0108 (8)	−0.0002 (7)
O2	0.0515 (11)	0.0702 (14)	0.0404 (10)	0.0242 (10)	0.0142 (9)	0.0166 (9)
C2	0.0241 (9)	0.0274 (10)	0.0231 (9)	0.0010 (7)	0.0109 (8)	0.0000 (7)
C3	0.0249 (9)	0.0317 (11)	0.0285 (10)	0.0016 (8)	0.0152 (8)	0.0017 (8)
C4	0.0266 (11)	0.0717 (18)	0.0401 (12)	0.0034 (11)	0.0208 (10)	0.0042 (12)
O1W	0.0283 (8)	0.0559 (13)	0.0346 (9)	0.0001 (8)	0.0066 (7)	−0.0048 (9)
C5	0.0236 (9)	0.0559 (15)	0.0263 (10)	0.0094 (10)	0.0100 (8)	0.0022 (10)
C6	0.0267 (10)	0.0378 (12)	0.0378 (11)	0.0020 (9)	0.0079 (9)	0.0014 (10)

### Geometric parameters (Å, º)

**Table:**

Cl1—C6	1.760 (3)	N2—H1N2	0.8600
Cl2—C6	1.770 (2)	N2—H2N2	0.8600
Cl3—C6	1.745 (3)	C3—C4	1.483 (4)
N1—C1	1.374 (3)	N4—H2N4	0.8600
N1—C3	1.292 (3)	N4—H1N4	0.8600
N2—C1	1.316 (3)	N5—H1N5	0.8600
N3—C2	1.325 (3)	C4—H4B	0.9600
N3—C1	1.348 (3)	C4—H4C	0.9600
N4—C2	1.319 (3)	C4—H4A	0.9600
N5—C2	1.361 (3)	C5—C6	1.557 (3)
N5—C3	1.355 (3)	O1W—H1O1	0.86 (4)
O1—C5	1.251 (3)	O1W—H2O2	0.78 (4)
O2—C5	1.212 (3)		
			
C1—N1—C3	115.80 (18)	C2—N5—H1N5	121.00
C1—N3—C2	115.8 (2)	C3—N5—H1N5	120.00
C2—N5—C3	119.06 (19)	C3—C4—H4A	109.00
N1—C1—N2	116.02 (18)	C3—C4—H4B	110.00
N1—C1—N3	125.08 (19)	C3—C4—H4C	109.00
N2—C1—N3	118.9 (2)	H4A—C4—H4B	109.00
N3—C2—N4	120.1 (2)	H4A—C4—H4C	110.00
N3—C2—N5	121.50 (19)	H4B—C4—H4C	109.00
N4—C2—N5	118.3 (2)	O1—C5—O2	128.6 (2)
C1—N2—H1N2	120.00	O1—C5—C6	114.4 (2)
C1—N2—H2N2	120.00	O2—C5—C6	116.9 (2)
H1N2—N2—H2N2	120.00	Cl1—C6—Cl2	109.27 (13)
N1—C3—N5	122.7 (2)	Cl1—C6—Cl3	108.40 (15)
N1—C3—C4	121.2 (2)	Cl1—C6—C5	109.74 (15)
N5—C3—C4	116.1 (2)	Cl2—C6—Cl3	109.12 (12)
C2—N4—H2N4	120.00	Cl2—C6—C5	108.12 (17)
C2—N4—H1N4	120.00	Cl3—C6—C5	112.16 (16)
H2N4—N4—H1N4	120.00	H1O1—O1W—H2O2	107 (3)
			
C3—N1—C1—N2	177.8 (2)	C3—N5—C2—N4	177.2 (2)
C3—N1—C1—N3	−2.4 (3)	C2—N5—C3—N1	0.4 (3)
C1—N1—C3—N5	1.3 (3)	C2—N5—C3—C4	179.3 (2)
C1—N1—C3—C4	−177.5 (2)	O1—C5—C6—Cl1	−59.0 (3)
C2—N3—C1—N1	1.6 (3)	O1—C5—C6—Cl2	60.1 (3)
C2—N3—C1—N2	−178.58 (19)	O1—C5—C6—Cl3	−179.51 (19)
C1—N3—C2—N4	−178.1 (2)	O2—C5—C6—Cl1	122.4 (2)
C1—N3—C2—N5	0.3 (3)	O2—C5—C6—Cl2	−118.5 (2)
C3—N5—C2—N3	−1.3 (3)	O2—C5—C6—Cl3	1.9 (3)

### Hydrogen-bond geometry (Å, º)

**Table:**

*D*—H···*A*	*D*—H	H···*A*	*D*···*A*	*D*—H···*A*
N5—H1*N*5···O1^i^	0.86	1.79	2.652 (3)	178
N2—H1*N*2···O1*W*^ii^	0.86	2.03	2.886 (3)	174
N2—H2*N*2···N1^iii^	0.86	2.21	3.071 (3)	174
N4—H2*N*4···N3^iv^	0.86	2.18	3.034 (3)	173
N4—H1*N*4···O1*W*^v^	0.86	2.22	2.834 (3)	128
O1*W*—H1*O*1···O1	0.86 (4)	1.97 (4)	2.835 (3)	176 (3)
O1*W*—H2*O*2···O2^vi^	0.78 (4)	1.97 (4)	2.741 (3)	173 (3)

Symmetry codes: (i) −*x*+1/2, *y*−1/2, −*z*+3/2; (ii) −*x*, −*y*+1, −*z*+1; (iii) −*x*, *y*, −*z*+1/2; (iv) −*x*, *y*, −*z*+3/2; (v) *x*, −*y*+1, *z*+1/2; (vi) −*x*+1/2, *y*+1/2, −*z*+3/2.
